# Questionnaire Development to Assess Risk Factors for Environmental Diseases of Children in Daejeon

**DOI:** 10.3390/jcm13154552

**Published:** 2024-08-04

**Authors:** Ji-Hye Oh, Il-Whan Choi, Jae-Eun Shim, Seock-Yeon Hwang

**Affiliations:** 1 Department of Biomedical Laboratory Science, Daejeon University, Daejeon 34520, Republic of Korea; ohjihae14@dju.kr; 2 Department of Environmental Engineering, Daejeon University, Daejeon 34520, Republic of Korea; 170cm80kg@dju.kr; 3 Department of Food and Nutrition, Daejeon University, Daejeon 34520, Republic of Korea; jshim@dju.kr

**Keywords:** allergic disease, environmental disease, prevalence, South Korea

## Abstract

**Background**: Allergic diseases are common in children and adolescents. It is important to assess the prevalence and risk factors of environmental diseases to implement tailored countermeasures. **Methods**: This questionnaire study investigated factors associated with environmental diseases in elementary school children with an environmental disease from 150 households in Daejeon Metropolitan City, South Korea in 2021. **Results**: The participants comprised 55.7% girls and 44.3% boys, and the mean age was 10.1 years with an even age distribution. The typical risk factors observed were the type of roads nearby, the presence of mold or stains within the residence, pet ownership, and frequency of indoor ventilation and cleaning. Notably, 73.2% of the households had an eight-lane road nearby, 40.2% reported leaks, stains, or mold within their homes during the past year, and 37.1% ventilated their homes for less than 30 min. After education on preventing and managing environmental diseases, significant changes were observed in bedding washing frequency, average ventilation duration per session, and duration of humidifier usage (*p* < 0.05–0.001), with improvements in lifestyle. **Conclusions**: Our study can be used as a reference for expanding indoor air quality control education for parents with children with an environmental disease and providing tailored environmental consultations.

## 1. Introduction

Allergic diseases are the most common chronic disease in children and adolescents worldwide and impact the daily living and quality of life of patients [1]. Particularly, atopic dermatitis (AD) is a common chronic inflammatory skin condition affecting 15–25% of children worldwide, with a similar prevalence rate among children in the Republic of Korea [2]. Approximately 85% of all patients with AD are children aged ≤ 5 years, and the condition persists through adulthood in 25% of these patients, impairing quality of life and causing sleep deprivation due to extreme itching [3,4]. The exact cause and pathogenesis of AD remain unclear, but environmental, immunologic, and genetic factors and epidermal barrier dysfunction have been proposed as etiologic factors [4]. AD involves a repeated cycle of improvement and exacerbation over a prolonged period, and failure to seek aggressive treatment or avoid exacerbating factors leads to asthma and rhinitis as the child grows, with an elevated risk for the allergic march [1,3]. In addition, 60% of patients have a relapse, and some patients reportedly experience allergic march, such as asthma and allergic rhinitis [4]. However, AD is one of the most misdiagnosed conditions in the Republic of Korea, resulting in unnecessary and wrong treatment, impaired quality of life, and increased healthcare costs, highlighting the need to study its prevalence and risk factors [3,5].

The global prevalence of AD was found to be 2.6% (95% uncertainty interval (UI) 1.9–3.5), affecting 204.05 million people. Additionally, the prevalence of AD is increasing in most countries, including Asia. For example, in the case of Taiwan, it was reported that the rate increased from 2.4% in 1996 to 4.0% in 2015. In terms of age, a similar increase in lifetime prevalence was observed in patients of similar age: 13–14 years in Taiwan and China, 7–15 years in Japan, and 12–15 years in Korea. Nevertheless, epidemiological data are known to be lacking in 41.5% of countries around the world.

According to a 2019 National Health Insurance Service report, Daejeon Metropolitan City has an AD incidence of 25.3 per 1000 population, higher than the national average among metropolitan cities with 18.7 per 1000 population. Particularly, most cases of AD occur at or under the age of 5 years. Considering that children in this age group spend most of their time indoors, such as in-home or group facilities, managing the indoor environment is speculated to be especially crucial among other environmental factors [3]. Consequently, a study investigated the impact of indoor substances, such as house dust mites and indoor air pollutants, on AD in young children, revealing that improving indoor air quality can help alleviate AD symptoms [6].

Indoor air quality varies widely depending on the type of facility and maintenance (e.g., ventilation frequency and pollutant control facilities) and type of indoor activities (e.g., cooking, smoking, and presence of pets). Compared to the general ambient air quality, ongoing monitoring and administrative management at the state and local government levels, including guidance and inspections, are challenging, leading to numerous limitations in developing comprehensive and systematic management strategies [7,8]. In other words, various indoor air pollutants may affect the health of vulnerable populations, such as children, older adults, and patients with pre-existing conditions such as allergic diseases [3]. Despite these findings, there is still no clear evidence regarding the causal relationship between indoor and outdoor air pollutants and AD in the Republic of Korea and abroad [9]. Therefore, in this study, we attempted to identify risk factors by investigating individual lifestyle habits and indoor living environment factors among children suffering from environmental diseases living in 150 households in the Daejeon area. Through this, we recognize the need to identify worsening factors based on more evidence by considering the various environmental conditions of individual patients and intend to use these as data to help establish continuous monitoring and management plans at the local government level.

## 2. Materials and Methods

### 2.1. Study Population and Protocol

Children recruited for the “Living Lab Platform Building for Environmental Diseases” project in Daejeon Metropolitan City in 2021 were considered for this study. The Living Lab Platform is a project to share problems of environmental diseases in all areas of Daejeon Metropolitan City and to establish industry/academia/research collaboration and comprehensive care support to derive solutions. Our survey was conducted among elementary school children of 150 households in Daejeon Metropolitan City with an environmental disease. A trained surveyor administered the survey via an online platform due to coronavirus disease. 

Subjects were recruited with the support of Daejeon Metropolitan City and Daejeon Education Office, and 150 patients were finally selected from the 300 who applied. The 150 subjects were selected according to district (Dong-gu, Jung-gu, Seo-gu, Yuseong-gu, Daedeok-gu), low-rise/high-rise (below 5 floors/above 5 floors), disease (atopy, allergic rhinitis, asthma), and housing type (detached house, townhouse, apartment).

We investigated the indoor risk factors of 88 patients with AD, 109 with allergic rhinitis, and 9 with asthma (conditions could overlap). Furthermore, as this study was conducted with elementary school children, a vulnerable population, we first obtained approval from the Institutional Review Board at Daejeon University (040647-202106-HR-017-02) and administered the questionnaires considering the participants’ rights. Written informed consent was obtained from the parents/guardians of the patients.

### 2.2. Survey Items

We developed the questionnaire based on the key items in the International Study of Asthma and Allergies in Childhood (ISAAC) questionnaire. We included questions on basic demographic information (grade level and sex), living environment, current health status, personal characteristics, disease history, and awareness of environmental diseases. The internal validity of the items was evaluated by obtaining advice from a food science professor, an environmental engineering professor, and the president of the Korean Atopy Forum.

### 2.3. Training for the Prevention and Management of Environmental Denitrification

Children and families with environmental diseases (atopic dermatitis, asthma, allergic rhinitis) in Daejeon Metropolitan City were educated about prevention and management methods through special lectures by experts (dermatologists, professors of Oriental medicine, nutritionists, and psychological counselors specializing in children). A total of three sessions were held at one-month intervals, and a survey on the prevalence of environmental pollution was conducted after each session.

Correlation analysis was performed to determine the differences in housing and environmental management before and after the environmental disease education program.

### 2.4. Statistical Analyses

To analyze participant characteristics, categorical variables are presented as percentages and frequencies, and continuous variables as means and standard deviations. To investigate the possible correlation between environmental diseases and demographic characteristics (sex ratio) of the study participants, cross-tabulation analysis was performed, and statistical significance was determined using Fisher’s exact test according to the expected frequency of less than 5. Changes in awareness and lifestyle after environmental disease education were analyzed using a paired samples t-test, and the results are presented as 95% confidence intervals (CIs). All statistical analyses were performed using IBM SPSS Statistics Ver 26.0 (IBM Corp., Armonk, NY, USA), and a *p*-value < 0.05 was considered statistically significant.

## 3. Results

### 3.1. Participant Characteristics

Elementary school children with an environmental disease from 150 households were surveyed. There were more girls (*n* = 54, 55.7%) than boys, and the mean age was 10.1 years. The most common environmental diseases were allergic rhinitis (77.3%) and atopic dermatitis (56.7%). When comparing the sex ratio, women accounted for the largest proportion of atopic dermatitis (67.3%), while allergic rhinitis was equally represented by both sexes. In the association of atopic dermatitis by sex ratio, the expected frequency less than five accounted for 18.62% of all cells, so Fisher’s exact test rather than Pearson’s chi-squared test was used to determine the probability of significance, and *p* = 0.008 showed an association between the response categories of having atopic dermatitis by sex ratio. The areas of residence were Seo-gu (36.1%), Jung-gu (21.6%), Dong-gu (16.5%), Daedeok-gu (14.4%), and Yuseong-gu (11.3%). The most common type of home was apartment (77.3%), and 38.1% lived on floor four or lower floors. Most participants lived in a residential area (94.9%), and 73.2% had an eight-lane road in front of their homes (Table 1 and Table 2).

Regarding past residences, 43.3% have lived in a different region in the past, with Daejeon (72.8%) being the most common, followed by Chungnam (9.1%) and Gyeonggi Province (5.5%). The duration of residence was below 6 years in 41.9% and below 9 years in 30.9%. Of those who had lived in the Daejeon Metropolitan City previously, the most common district (gu) was Jung-gu (27.5%) and Dong-gu (25.0%), followed by Seo-gu, Daedeok-gu, and Yuseong-gu.

### 3.2. Analysis of the Association between Environmental Diseases and Indoor Environmental Factors

#### Factors Related to the Living Environment

Most households used a gas boiler heating system (81.4%). The most common floor material was polyvinyl chloride, while the most common wall finishing material was wallpaper (94.8%). Approximately 8.2% of the households had had their homes remodeled in the past six months, and relatively high percentages of households had leakage (19.6%) and stains or molds (40.2%) in the past year. Other factors that may contribute to the prevalence of environmental diseases were surveyed. In addition, 30.7% of the households owned pets, such as birds, cats, and dogs, and 9.8% owned things that may cause fur or dust, such as carpets and dolls (Table 3 and Table 4).

### 3.3. Factors Affecting Indoor Hazardous Factors and Air Pollution

A relatively high percentage of households (27.8%) had a smoker in their family, and 3.6% stated that the smoker had smoked while children were around. Regarding home cleaning frequency, 51.5% were cleaned every day, while 35.4% were at least twice a week. Regarding bedding laundry frequency, 39.2% washed their bedding once every two weeks, 29.9% washed bedding once every month, and 13.4% claimed that they did not wash their bedding frequently. The most common indoor ventilation frequency, which has the greatest impact on indoor air quality, was 1–2 times/day (42.3%), and 37.1% of the families ventilated their homes for an average of 10–30 min each time (Table 5).

### 3.4. Participant Personal Characteristics and Disease History

Regarding personal disease history, asthma, allergic rhinitis, and AD, the percentages of participants diagnosed by a physician were 13.4%, 77.3%, and 56.7%, respectively. Current prevalences were 5.2%, 68.0%, and 42.3%, respectively (Table 6, Table 7 and Table 8).

The prevalence of AD and asthma symptoms in the past 12 months was surveyed using a standardized questionnaire developed by ISAAC and translated and validated in Korean. All 13.4% of patients who currently have asthma responded “yes” to the question, “In the past 12 months, have you heard a wheezing or whistling sound when breathing during or after exercise?” The symptoms were most common in January. All 42.3% of patients who currently have AD answered “yes” to the question, “In the past 12 months, have you had itchy skin rashes?” The symptoms were most common in April and May (11.4%) and least common in January and August (1%) (Table 9).

### 3.5. Analysis of Changes in Living Environment Management after Education on Environmental Diseases 

#### Living Environment

Changes in the management of living environments after providing education on environmental diseases were analyzed using a paired samples t-test. The results indicated changes in bedding laundry frequency, average duration of ventilation per session, and humidifier use. The responses were scored with “never” as 1 point and “1 time/week” as 5 points, and the score was converted to a 100-point score. The mean bedding laundry score was 61.33 before education and significantly improved to 69.92 after education at *p* < 0.05 (t= −2.117, *p* = 0.038). The average duration of ventilation per session was scored with “never” as 1 point and “≥1 h” as 5 points and converted to a 100-point score. The results indicated a significant improvement from 41.41 before to 59.76 after education at *p* < 0.001 (t = −5.624, *p* = 0.000). Duration of humidifier use was scored with “never” as 1 point and “≤ 24 h” as 5 points and converted to a 100-point score. The results indicated a significant improvement from 13.67 before education to 22.26 after education at *p* < 0.001 (t = −3.701, *p* = 0.000) (Table 10).

## 4. Discussion

According to a Korea Ministry of Health and Welfare report, failure to properly manage allergic diseases in childhood and adolescence may lead to the allergic march and progression to more severe disease in adulthood, thereby incurring financial burden and loss [10]. Particularly, people in modern society spend 80–90% of their time indoors, and prolonged exposure to contaminated indoor air in mass-gathering facilities as well as in the home environment among infants, young children, and postpartum women, who have relatively weaker immune systems, could trigger respiratory illnesses and AD due to bacterial proliferation, thus highlighting the importance of proper indoor air quality management [11].

This study showed that the type of roads near the home, mold and stains within a residence, the presence of pets, indoor ventilation, and cleaning frequency were risk factors among children with an environmental disease. In addition, appropriate responses to risk factors were not being implemented, suggesting a lack of awareness and poor management methods for environmental diseases and the need for improvement in such areas. Approximately 73.2% of our study households were in areas close to an eight-lane road, and studies have reported that such residential conditions are associated with high levels of biological hazards that contribute to environmental diseases (increased indoor total airborne bacteria, total mold fungi, *Dermatophagoides pteronyssinus*, and *Dermatophagoides farinae*). Furthermore, such environments have also been linked to a high risk of exposure to muconic and hippuric acids and metabolites of volatile organic compounds [12]. Additionally, there is a substantial body of research suggesting that exposure to such pollution is associated with an approximately 40% increased risk of developing asthma and autism in early childhood [13,14].

A high percentage of households (40.2%) stated that they had leakage, stains, or molds in their homes during the past year. According to the report from the Environmental Health Center for Atopic Diseases at Samsung Medical Center, children in homes affected by water damage have a 15-fold higher risk of exacerbation of AD [15]. Additionally, mold, which adversely affects AD, was found to be up to five times more abundant in households with confirmed water damage than in unaffected homes. Furthermore, unlike house dust mites, pollen, and animal fur or hair, mold germinates and proliferates within respiratory epithelial cells once it enters the respiratory system, triggering excessive host defense mechanisms and causing allergic reactions. Tiny, fragmented mold particles can penetrate the lower respiratory tract and cause inflammation and airway obstruction, elevating the risk of AD as well as asthma and allergic rhinitis. Moreover, a US study indicated that elevated levels of mold antigens, in conjunction with other allergens such as house dust mites, cockroaches, and cat allergens, were linked to elevated levels of specific IgE antigens in the body [16]. Consequently, there is a need to educate families on improving their indoor environments by placing hygrometers in their homes and preventing the accumulation of water or excess humidity during the rainy season [17].

The most common indoor ventilation frequency and duration among households with children with an environmental disease were 1–2 times/day (42.3%) and 10–30 min (37.1%), respectively. Adequate indoor air ventilation during days with low fine dust levels to maintain optimal indoor carbon dioxide concentration and temperature has been reported to help ameliorate reduced skin moisture in AD [6]. Furthermore, insufficient ventilation can lead to indoor air pollution and facilitate the proliferation of microorganisms such as mold and dust mites [18]. Hence, it is recommended to fully ventilate homes for at least 30 min, three times a day.

As allergic symptoms significantly impair quality of life and disrupt individual daily living, proper management of daily environmental conditions is crucial and requires relevant education [19]. After providing a three-session education about environmental disease prevention, we observed significant improvements in lifestyle scores. The greatest changes were observed in indoor cleaning frequency, duration of ventilation per session, and indoor ventilation frequency. We speculate that recognizing the need for these practices through education and engaging in efforts to decrease dust in the indoor environment led to these significant outcomes. Our results can serve as foundational data for expanding indoor air quality control education for parents with children with an environmental disease and providing tailored environmental consultations.

This study had limitations. First, we could not compare the relative contamination in vulnerable households directly, as indoor environments in households without children but with an environmental disease were not assessed. Second, the study population was 100 households, which is not representative of the entire population with environmental disease in Daejeon Metropolitan City; thus, an expansion of the study population in future studies is required. Third, analyzing the prevalence of environmental diseases and risk factors solely based on a questionnaire may have had limitations. Accordingly, correlation analysis involving surveys is necessary via biological monitoring and evaluation of allergy-related components in the body. This is expected to increase accuracy in identifying risk factors for environmental diseases.

Furthermore, the existing data are limited to local observations with a relatively small number of participants. Plans are in place to enhance the quality of the data by conducting additional studies over the next five years. These will focus on monitoring indoor air quality and identifying prevalence factors in households with environmental diseases. By diversifying the assessment methods and increasing the population sample for data analysis, it is anticipated that the management of vulnerable populations will become more straightforward, and the prevalence of environmental diseases will decline.

## Figures and Tables

**Table 1:** Participants’ general characteristics.

	Category	*n* (%)		Category	*n* (%)
Sex	Male	43 (44.3%)	Grade level	1st	25 (25.8%)
				2nd	15 (15.5%)
				3rd	25 (25.8%)
	Female	54 (55.7%)		4th	7 (7.2%)
				5th	9 (9.3%)
				6th	16 (16.5)
Area of residence	Dong-gu	16 (16.5)	Type of home	Single home	5 (5.2)
	Jung-gu	21 (21.6)		Townhome	14 (14.4)
	Seo-gu	35 (36.1)		Apartment	75 (77.3)
	Yuseong-gu	11 (11.3)		Studio	1 (1.0)
	Daedeok-gu	14 (14.4)		Commercial–residential property	2 (2.1)
Environment near residence	Industrial area	1 (1.0%)	8-lane road nearby	Yes	71 (73.2%)
	Residential area	92 (94.9%)		No	26 (26.8%)
	Commercial area	3 (3.1%)			
	Redevelopment area	1 (1.0%)			

**Table 2:** Prevalence of environmental diseases by sex.

Category	Environmental Disease (Allergic Diseases)	*n*		Fisher Exact Test (*p*)
		Man	Woman	
Atopic dermatitis	Not available	25 (25.8%)	17 (17.5%)	0.008 **
	Available	18 (18.6%)	37 (38.1%)	
Allergic rhinitis	Not available	6 (6.2%)	16 (16.5%)	0.055
	Available	37 (38.1%)	38 (39.2%)	
Asthma	Not available	35 (36.1%)	49 (50.5%)	0.180
	Available	8 (8.2%)	5 (5.2%)	

** *p* < 0.01: significant difference by Fisher’s extract test

**Table 3:** Heating systems and finishing materials in homes.

	Category	*n* (%)		Category	*n* (%)
Heating system	Gas boiler	79 (81.4)	Floor material	PVC	57 (58.8)
	Central boiler	18 (18.6)			
				Paper	3 (3.1)
	Coal boiler (Underfloor heating)	0 (0.0)			
				Ceramic	3 (3.1)
				Wood	29 (29.9)
	Central heating	0 (0.0)			
				Marble	2 (2.1)
	Firewood, wood chips, straw	0 (0.0)			
				Floor	3 (3.1)
Wall finishing material	Wallpaper	92 (94.8%)	Interior remodeling in the past 6 months	Yes	8 (8.2)
	Paint	3 (3.1%)		No	89 (91.8)
	Brick	1 (1.0%)	Leakage within the home in the past 1 year	Yes	19 (19.6)
	Tile	4 (4.1%)		No	78 (80.4)
	Wood	0 (0.0%)	Stain or mold in the past 1 year	Yes	39 (40.2)
	Marble	1 (1.0%)		No	58 (59.8)

Abbreviation: PVC, polyvinyl chloride.

**Table 4:** Ownership status.

Category	Frequency	(*n*)	Percentage	(%)
Gas and oil heater	87		9.7	
Briquette heater	96		10.7	
Wall heater	53		5.9	
Humidifier	35		3.9	
Air conditioner	70		7.8	
Ownership status Carpet	77		8.6	
Large stuffed dolls	62		6.9	
Child’s own bed	26		2.9	
Bird	93		10.3	
Cat	95		10.6	
Dog	88		9.8	

**Table 5:** Home cleaning, laundry, and indoor ventilation frequency.

	Category	*n*	Percentage (%)		Category	*n*	Percentage (%)
Home cleaning frequency	Every day	50	51.5%	Bedding laundry frequency	1 time/week	17	17.5%
	≥2 times/week	34	35.4%		1 time/2 weeks	38	39.2%
	1 time/week	11	11.3%		1 time/ month	29	29.9%
	1 time/2 weeks	1	1.0%		Not frequently	13	13.4%
	1 time/3 weeks	1	1.0%				
Category		*n* (%)		Category		*n* (%)	
Indoor ventilation frequency	5 times/day	27 (27.8)		Mean duration of each ventilation	<10 min	5 (5.2)	
	3~4 times/day	20 (20.6)			10–29 min	36 (37.1)	
	1–2 times/day	41 (42.3)					
	2–3 times/week	6 (6.2)			30–59 min	25 (25.8)	
	≥1 time /week	3 (3.1)			≥1 h	31 (32.0)	

**Table 6:** Personal characteristics and disease history (asthma).

	Category		Frequency (*n*)	Percentage (%)
Personal characteristics and awareness (diagnosis of environmental disease)	Asthma (physician’s diagnosis)	No	84	86.6
		Yes	13	13.4
	Asthma (age at first diagnosis)	2 years	2	2.1
		3 years	1	1.0
		4 years	3	3.1
		6 years	2	2.1
		7 years	1	1.0
		8 years	1	1.0
		10 years	2	2.1
	Currently has asthma	No	92	94.8
		Yes	5	5.2
	Currently being treated	No	92	94.8
		Yes	5	5.2

**Table 7:** Personal characteristics and disease history (allergic rhinitis).

	Category		Frequency (*n*)	Percentage (%)
Personal characteristics and awareness (diagnosis of environmental disease)	AD (physician’s diagnosis)	No	42	43.3
		Yes	55	56.7
	AD (age at first diagnosis)	1 year	15	15.5
		2 years	8	8.2
		3 years	5	5.2
		4 years	5	5.2
		6 years	1	1.0
		7 years	1	1.0
		8 years	3	3.1
		9 years	3	3.1
		11 years	1	1.0
		13 years	1	1.0
	Currently has AD	No	56	57.7
		Yes	41	42.3
	Currently being treated	No	79	81.4
		Yes	18	18.6

**Table 8:** Personal characteristics and disease history (AD).

		Category	Frequency (*n*)	Percentage (%)
Personal characteristics and awareness (diagnosis of environmental disease)	AD (physician’s diagnosis)	No	42	43.3
		Yes	55	56.7
	AD (age at first diagnosis)	1 year	15	15.5
		2 years	8	8.2
		3 years	5	5.2
		4 years	5	5.2
		6 years	1	1.0
		7 years	1	1.0
		8 years	3	3.1
		9 years	3	3.1
		11 years	1	1.0
		13 years	1	1.0
	Currently has AD	No	56	57.7
		Yes	41	42.3
	Currently being treated	No	79	81.4
		Yes	18	18.6

Abbreviation: AD, atopic dermatitis.

**Table 9:** Survey of asthma and AD prevalence using the ISAAC questionnaire.

Category		Frequency (*n*)	Percentage (%)
Wheezing or stridor in the past 12 months	Yes	5	5.2
	No	92	94.8
Type of symptom onset (asthma)	January	2	2.1
	July	1	1.0
	Not applicable	94	96.9
Category		Frequency (*n*)	Percentage (%)
Itchy skin rash in the past 12 months	Yes	55	56.7
	No	42	43.3
Time on symptom onset (AD)	1	1	1.0
	2	3	3.1
	3	3	3.1
	4	5	5.2
	5	6	6.2
	6	3	3.1
	7	4	4.1
	8	1	1.0
	10	4	4.1
	11	2	2.1
	12	3	3.1
	Not applicable	62	63.9

**Table 10:** Comparison of lifestyle before and after environmental disease education.

Category		Descriptive Statistics			Confidence Interval (C.I) 95%		t (*p*)
		*N*	Mean	Standard Deviation	Lower	Upper	
Bedding laundry frequency	Before	64	61.33	29.94	−18.53	2.12	−2.117 (0.038) ^*^
	After	64	69.92	28.84			
Daily average duration of indoor ventilation	Before	64	41.42	21.00	−25.33	−11.39	−5.264 (0.000) ^***^
	After	64	59.76	21.65			
Duration of humidifier use	Before	64	13.67	23.12	−24.06	−7.19	−3.701 (0.000) ^***^
	After	64	22.26	27.14			

Values are presented as mean ± standard deviation (95% confidence interval). * *p* < 0.05, *** *p* < 0.001: significant difference by paired *t*-test.

## Data Availability

All data generated or analyzed during this study are included in this published article.
